# From spatial perception to referential meaning: convergent image schemas in the music of and texts about Beethoven’s piano sonatas

**DOI:** 10.3389/fpsyg.2024.1497557

**Published:** 2024-11-25

**Authors:** Mihailo Antović, Vladimir Ž. Jovanović, Mladen Popović

**Affiliations:** Faculty of Philosophy and Center for Cognitive Sciences, University of Niš, Niš, Serbia

**Keywords:** image schema, metaphor, language, music, Beethoven, spatiality

## Abstract

This paper approaches the connection between musical constructs and visuo-haptic experience through the lens of the cognitive-linguistic notion of the “image schema.” The proposal is that the subconscious inference of spatial and haptic schematic constructs in music, such as vertical movement, will motivate their equally common occurrence in the language about that music, irrespective of the fact that this language never describes the musical structure in a one-to-one fashion. We have looked for five schemas in the scores for the first ten piano sonatas by Ludwig van Beethoven and three famous analytical and pedagogical texts about them: force, indicating changes in musical dynamics and referential invocation of power-related terms in the books; path, identifying vertical movement in the music and suggestions of upward- or downward motion in the texts; link, suggesting the presence or absence of musical slurs and references to attachment or detachment in the language; balance, indicating the loss and regain of consonance in the harmony and invocation of lost and recovered stability in the verbal semantics; and containment, allocating the nonharmonic tones that “belong” to their resolving notes in the scores and referring to physical or metaphorical enclosed areas in the texts. Results of the corpus analysis suggest the following conclusions: musical schemas outnumber linguistic ones sevenfold; moderate schema strengths are typical of both language and music; predominant valences are shared by language and music in three schemas out of five; hierarchies of five schemas by strength differ, though the strongest schemas are mostly shared. Yet the central finding is that the correlations between each schema pair for music and language, by scalarity and valence, are total. This implies that (1) schemas operate as semantic building blocks irrespective of the external “symbolical form” in which they are realized and (2) scalarized image schema complexes perceived in one cognitive mode may motivate the emergence of a corresponding number of the same complexes in another.

## 1 Introduction

If a musical excerpt seems to listeners to “move” all the time, will this movement be equally frequently found in the analytical language about this music? If a score is packed with ascending note sequences, forte dynamics, slurs instructing the performer to play legato, unstable harmonies, or appoggiaturas implicitly “belonging” to their resolving notes, will concepts grounded in such (upward) paths, forces, links, (dis)balances and containments be found in pedagogical texts about this music, as well?

The purpose of the present paper is to investigate five types of conceptual connections between musical and spatial/haptic structure indicated in small capitals above, using the first ten piano sonatas by Beethoven as a test case. As cognitive linguists also interested in what some have called the “cognitive semantics” (Antović, 2009a; Brandt and do Carmo, 2015) or “cognitive linguistics” of music (Wallmark and Kendall, 2018) we aim to approach these connections through the abstract notion of image schemas (Johnson, 1987; Oakley, 2007). Namely, cognitive linguistics purports that meaning arises from early bodily interaction, e.g., climbing a flight of stairs to reach one’s favorite toy, which later becomes mentally internalized in a schematic form (e.g., ascending along a path to reach a goal). Such a schematization is immediately associated with affect (elevation with a desired outcome breeding positive valence), and finally generalized onto abstract domains, usually in the form of metaphor (the yearning to “move upward along a social ladder” or indeed the satisfaction upon hearing “a musical scale reaching the upper tonic”). Schemas are thus relevant to the problem of conceptual development in psychological inquiry, and manifested in constructing and interpreting meaning sequences in language and music. Our primary aim is to employ the same approach to studying music and language in an attempt to learn how the image schema theory could help uncover any parallels in structuring musical cognition and the language used directly in reference to the corresponding music. In other words, this research is focused on discovering how music and language encode the same emotional-cognitive content based on image schemas as the foundational building blocks of meaning. The results of this study may then serve to back theories claiming that there is an abstract, schematic basis behind concept construction, providing a strong case for the idea that the inference of schematic, predominantly spatial structure in one mode sparks the equally abundant use of the same schemas in another.

The theoretical concept of the image schema was advanced by both linguists and developmental psychologists, namely Cienki (1997), Gibbs et al. (1994), Gibbs (2005), Johnson (1987, 2005, 2007), Lakoff (1987, 1990), Lakoff and Turner (1989), Mandler (1992, 2004, 2012), Mandler and Cánovas (2014), Oakley (2007) and others. An image schema is a structure based on our immediate sensorimotor and perceptual experience coming from the interaction with the outside world, primarily on the basis of spatial relations such as in-out, near-far, up-down, but also on object manipulation, movement through space, etc. It is not purely perceptual, as it serves to build consciously accessible concepts (Mandler, 2004). The experience that we have from the physical world is re-engaged in helping us construct notions in the domain of the non-physical, reasoning and cognition through image-schematic structures (Johnson, 2007: 139–141). Communication that can make use of image-schema based concepts involves many codes such as language, music or vision. The distinction between center and periphery, for instance, underlies linguistic metaphors such as “This is the central point in our discussion,” appreciation of musical structures such as the tonic as the “center toward which all the chords gravitate,” or visual presentations, e.g., the positive affective load of the face of Christ as the vantage point in da Vinci’s The Last Supper (Antović, 2021). The same cross-modal logic appears to hold for numerous other image schemas, such as verticality, force, link, balance, blockage, source-path-goal, to name but a few. Language as a specific system of form-meaning-based symbolism has been studied in this respect the most, as it relies significantly on image-schematic building blocks by means of which reference is made to phenomena from the extralinguistic reality. In the seminal works on image schemas referred to above, these “experience distillers” (Oakley, 2007) are perceived as mental representations in our memory or gestalts composed of few interrelated parts coming across perhaps not as “extremely skeletal images” (Turner, 1991: 57), but rather as conceptual drafts immersed in “the felt qualities of our experience, understanding and thought” (Johnson, 2005: 31). Being internally structured patterns, they exhibit considerable flexibility, which is manifest both in the transformations they undergo and the interaction processes with other image schemas they engage in, all of which […] closely relates to perceptual (gestalt) principles (Hampe, 2005b). Not only have individual image schemas such as center-periphery been viewed as gestalt structures, but also different possible groupings of image schemas which tend to be experienced interdependently have been considered experiential gestalts (Cienki, 1997: 7–9).

Ever since the origination of the image schema theory in the late eighties, there has been experimental work attempting to corroborate the idea that schemas make an important part of human cognition and language. As Gibbs et al. (1994) and Gibbs (2005) report, the interest of a number of case studies was centered around the idea that understanding sentences and lexical items involved an underlying image-schematic structure which again relied on embodied meaning construal in the course of the online linguistic expression processing. For instance, Klatzky et al. (1989) suggested that embodied actions of hand shaping to stand for the verbs “pinch” and “clench” could influence sensibility judgments on the collocations “aim a dart” (sensible) or “close a nail” (not sensible) in such a way as to enhance the speed of reaction, whereas this was not the case with verbal primes. Zwaan et al. (1995) considered the impact of varied time adverbials, where those with implied longer period of time, such as “an hour later,” extended the reaction time as compared to the sentence with “a moment later,” indicating a connection between certain mental imprints of experience and language processing. Having conducted research on the basis of sentence pairs with aspect-marked verb phrases, Carreiras et al. (1997) showed that language units with aspectual distinction for progression were easier for processing than the ones unmarked or with indefinite aspect. Also based on sentential meaning interpretation, another study investigated the link between image schemas represented by pictures, matching and mismatched in terms of orientation, and improved understanding of sentences with verbally corresponding expressions (Stanfield and Zwaan, 2001). The results confirmed that the response times were much shorter when the picture and the sentence matched in orientation. In Richardson et al. (2003), it was shown how image schemas are engaged in interpreting concrete and abstract verb meanings through pairing images representative of four image schemas and the best verbal equivalent in the language.

When it comes to metaphorical language studies, among the pioneering books was Goosens et al. (1995), which provides plenty of examples on how different image schemas help shape up metaphorical concepts, e.g., “shut one’s mouth,” “take one’s breath away” (container), “blowing off steam” (force, containment, balance), “thrust something down one’s throat” (path, container), etc. (Goosens et al., 1995: 45–49). They appear to do so by presenting the glue that puts together the embodied actions with language expressions. Mori (2019) studied image-schema transformations that affected the metaphorical extensions of the preposition “over,” while the frame was also used even in a linguistic study of psychological features of a literary character in The Great Gatsby (Zhou et al., 2023). These studies have inspired the central idea of the present paper: that, similarly to the way in which gestural and pictorial information primes response times in language, schematic structures inferred from music may work as prompts for the frequent use of the same structures in language.

In connection with cognitive musicology, image schemas have been studied experimentally, in verbal descriptions of basic musical relations (e.g., distances between pitches, paths in scales, forces in changed dynamics) by Serbian and Romani children (Antović, 2009b) and US sighted and blind children (Antović et al., 2013). Suitability judgments, in the form of Likert-scale responses to preferred cross-modal stimuli, as made by young and adult musicians and nonmusicians, were also tested, suggesting an abstract structure beneath apparently different cross-cultural schematizations of musical scales (Antović et al., 2020). Theoretically, our group attempted to treat image-schema complexes (e.g., force, path, and link) as elements of generic spaces that motivate various output conceptual blends, e.g., a description of a staccato segment (+force, +path, −link) as “walking on tiptoes” (Antović, 2018). Yet there have been even broader proposals, with image schemas occasionally used as motivating constructs for entire music theories. This was the case with Saslaw (1996), who, among other things, opted to view musical modulations as instances of the source-path-goal schema. Furthering on this idea, Brower (2000) made a distinction between (intra-)musical and image schemas, e.g., center-periphery gestalts in harmonic relations working as broader nested containers. Finally, there have been authors proposing new image schemas that might operate only (or typically) in music, e.g., exertion (Cox, 2016) or oscillation (Malawey, 2010). In the third domain of research, authors have provided relevant music-analytical work, e.g., Johnson and Larson's (2003) appreciation of a Beatles song through the concept of “the spatial schema” which motivates musical movement, or Zbikowski’s (2008) analysis of the schematic nature of numerous musical elements, e.g., pizzicato suggesting the act of knocking in a Bach piece.

More recently, there have been developments in the attempt to expand on the notion in terms of image schema combinatorial potentials (Hedblom et al., 2015, 2016, 2019), as well as their scalarized individual manifestations (Antović et al., 2023). Differing to a considerable extent from any other approach used so far, this conception is based on the idea that image schemas in interaction normally build groupings in any more complex conceptualization (in both music and language) and are individually marked by the higher-order principle of *scalarity*, a discretized system of valences bringing a range of formalized intensities or distances to the schemas, thus decisively affecting the overall nature of the conceptualization. For instance, both pianissimo and fortissimo in music are instances of force, yet their intensities and valences differ. The same applies to the linguistic “He squeezed the button” and “He released the button,” which equally realize the force schema, yet with opposite valence. It has been shown by small-scale empirical research that any variation of the parameters changes the affective valence and arousal in participants’ cross-modal, referential descriptions. The annotation tags and scalarity valence marks used, as well as the procedure for identifying image schemas in language, were based on the Image schema identification and annotation protocol developed and verified by the SCHEMAS Group 2022 and presented in Figar and Veličković (2022), and Figar et al. (2024).

Based on some of the more exhaustive lists of image schemas proposed by Clausner and Croft (1999) and Evans and Green (2006), respectively, there are 41 specific image schemas outlined in the literature, but their total register “…has never constituted a closed set.” (Hampe, 2005: 2). On a more concrete level, image schemas could be exemplified by verticality and (upward) path in rising physical objects, musical scales, or psychological moods. Thus, for example, any perceived visual, musical or linguistic motion requires the coextension of at least force (needed to initiate the movement) and path (required for the agent to span a spatial range). Additionally, such combined forces and paths occur in various intensities, which as a rule motivate an affective interpretation of the phrase or event. If “troops [that, literally] go into Sievierodonetsk” exhibit an unmarked force and path (F+, P+), “troops [that, metaphorically] storm Sievierodonetsk” require much stronger scalarity: (F+++, P++). Arguably, all else being equal, the emotional reaction to the latter expression is significantly stronger. Similarly in music, an unmarked ascent through a fifth (F0, P+ up), is visibly “tamer,” and thus less affectively uplifting, than a fortissimo ascent through two or more octaves (F++, P+++ up) (Antović et al., 2023). Pursuing further the ideas promoted in Antović et al. (2023), Jovanović et al. (2024) have attempted to validate this approach to affective meaning construction/interpretation empirically in the journalistic register of political discourse. The research was meant to ascertain the application of this formalized approach to calculating affective meaning on a bilingual (English and Serbian), image-schema-annotated language corpus of almost half a million words, and establish ways toward any sustainable bias detection based on abstract formal-element processing. Grounding the findings of the study on statistical analysis, the authors concluded that despite the considerable common core regarding the image-schematic complexes and conceptualizations in general, finer differences could be found by assessing the emotional involvement and the affectiveness by the participants in a communication act. When the schemas marked for scalarity in higher values are engaged in conceptualizations, this could indicate higher levels of implication, since the higher the occurrence of the +valence schemas, the more affectively the communication is colored. This mechanism proved to be particularly sensitive when the topic was regarded, as in reference to war and dependent on the parameter of newspaper type.

Since image schemas have been envisaged as basically relational, gestalt-like conceptual primitives dominated by primarily spatial constructs with cross-modal significance, it is only understandable that their fundamental feature would be mirrored in the expression by the language of music. The sounds produced in the “empirical channel” would be evocative of spatiality rather than temporality in our perception. This thesis aligns nicely with oft-quoted theses in cognitive linguistics that conceptual construction is strongly based on spatial inferences (Mandler, 1992; Jackendoff, 2002). The proposal is equally clearly pointed out in the works of music theorists such as Larson (2004, 2012) who based his theory on the notion of “force dynamics” by the cognitive linguist Leonard Talmy (1988), but equally suggested that there is an analogy in the way we conceive of the flow in physical space when compared to the auditory space. A similar thesis can be found among philosophers working on music perception such as Di Stefano (2022: 175–176, 180) who studied the auditory-spatial correspondences both literally, in terms of source localization and metaphorically, by the conceptualization of auditory perception as movement. By the same token, the cognitive psychologist Hubbard (2017, 14–15) in his review basically added that the mental representation of music embodies a dynamic of motion, particularly with regard to musical gravity, magnetism and inertia, but also with the addition of momentum-like effects in music.

As part of a larger-scale research project dealing with conceptual construction in three different codes, namely music, language and visual cognition, the present inquiry focuses on the role of image schemas in underlying meaningful units and tries to establish the corresponding and differing elements in image schema manifestation, implication and combination within the ten piano sonatas by Ludwig van Beethoven. The main hypothesis is that the subconscious inference of image-schematic structure in music prompts the construction of a convergent schematic structure in language (the pertinent texts about the sonatas).

What is implied by the term “convergent” in this sense? The key objective is to investigate how two different semiotic systems would encode the same or relatively the same content with the signs at their disposal which do not entail one-to-one correspondence, as the basic units of neither music nor language would formally and semantically correspond. The unmatching and unmatchable nature of the basic units of the two modalities suggests that there would potentially be a considerable difference in the ways they encode the semantic content and suggest huge discrepancies and incommensurability between the two in terms of the type, frequency and positioning of the conceptual atoms. Yet we hypothesize that this discrepancy will not occur. Of course, we do not expect the absolute numbers of schemas in the musical scores and linguistic texts about them to be the same, nor do we anticipate that the textual material will follow the musical structure “note-by-note” or “phrase-by-phrase.” In fact, it might be the case that substantial parts of the linguistic discourse should not directly describe the musical structures from the sonatas at all. But this is precisely what makes the image schema construct so potentially powerful. Namely, if this obvious lack of total correspondence between the musical structure and the language describing it still results in some strong correspondences in the schematic structures of the two modes (e.g., the same overall prevalence of some schemas, valences, or intensities against others in music, and then in language), this would potentially suggest that the text writers have indeed been strongly “influenced” by the varying schematic structure of the music while writing their pieces. Yet, this may not have been the case due to the fact that the texts were simply “about” this music, but rather due to the book writers’ subconscious inference of our targeted musical schemas, which then functioned as a prompt, if not a full prime, for the authors to use them equally frequently in the texts. These expectations are defined more precisely in our hypotheses:

1. Due to the ontological difference between image schemas in musical and linguistic semantics (formal - structural - iconic in music and representational - referential - interpretive in language), there will be more instances of our five targeted schemas in music.
2. Unmarked and relatively marked schemas by intensity (e.g., F-, F--, F+, F++) will be significantly more prevalent than the strongly marked schemas (e.g., F--- or F+++) in both language and music. This will show that the intensity of affect suggested in the musical score matches the affect in the descriptions of said music. Likewise, if level two scalarity (e.g., F++) is as strong as level one (e.g., F+), this will correspond with the proverbial view of Beethoven’s music, and descriptions thereof, as quite energetic (e.g., loud, fast, with pronounced pitch spans).
3. The ratio of positive and negative valences on the scalarity continuum, per schema, will be the same in music and language (e.g., more forte articulation in the music will result in more “forceful” language about this music). This will suggest that, even though the absolute numbers of schemas are different, the predominant valences of schema-based structures in the music and the language about it are equal.
4. The hierarchy of schemas by frequency will be the same in music and language. In other words, if there are the most force schemas in language, followed by path, link, etc., such an ordering should be found in the music, too. This will prove that, even if the absolute numbers are different, the relative shares of the schemas viewed against one another in the two forms coincide.
5. Most importantly, there will be a correlation between changes in schema type, valence and scalarity taken together (e.g., if F++ schema numbers rise in music, they will tend to rise in the language about this music, too). This will suggest that, even if their absolute numbers are different, distributions of specific schema valences and scalarities are intrinsically interrelated in the music and the language about this music, to the point at which one may propose that the schematic structure of the music unconsciously primes the cognizer to use a corresponding structure in the text, too, even when this text is not directly descriptive of the musical structure.

The main objective of the paper is, therefore, to look for any convergences in the prevalence of schema combinations and intensities in the two cognitive modalities, suggesting that schemas may operate as significant semantic building blocks on an even more abstract level than so far postulated, that is, irrespective of the external “symbolical form” in which they are realized. A likely reason for this is the priming effect, where the inferred schematic structure from the music elicits a corresponding such structure in language, too.

## 2 Materials and methods

As the goal, specified in the hypotheses above, was to look for any convergences in the prevalence of image schemas in select pieces of classical music and reputable texts about those pieces, the present research took the form of a corpus study based on two types of sources: musical and linguistic.

The musical corpus comprised the first ten complete piano sonatas by Ludwig van Beethoven. These were selected as they represent some of the most well-known examples from the Western common-practice tradition. Similarly, such a choice naturally links to the first, programmatic study from our current research program, which analyzed the image-schematic structure of the beginning of this composer’s Sonata Op. 2 No.1 for the piano in F minor (Antović et al., 2023). An important additional reason for our such selection was that, having composed on the verge between the Classical and the Romantic periods, Beethoven remains universally acclaimed for both respecting the traditional musical form and breaking away from it toward a more intense characterization of musical affect (e.g., Hatten, 2004: 132). We needed the former to clearly delineate the musical schemas in the scores and the latter to still detect sufficient schema scalarization. Beethoven thus seemed like a logical choice for our purposes. The size of such a musical corpus that we compiled (10 sonatas, 35 movements, 6,621 measures, 89,261 notes) exceeds most comparable corpora used in analytical and musicological research on Beethoven available in the literature (which normally either focuses on individual sonatas, e.g., Ockelford, 2005 or appreciates opening movements only, e.g., Konz, 2012). Importantly, we analyzed all three or four movements per sonata. As these sections considerably differ in style, mood, and affect (based on musical factors such as tempo, dynamics, etc.), we were able to make a much more nuanced comparison of schema distribution than would have been the case with using first movements only.

As mentioned in the introduction, for the present purpose we were interested in five image schemas: path (P), force (F), link (L), containment (C), and balance (B). We located these in the musical material using suggestions from reputable sources in the literature (Saslaw, 1996; Brower, 2000; Zbikowski, 2008). In particular, we used our own programmatic article in this project (Antović et al., 2023), to additionally annotate *valence* and *intensity*, where each schema was scalarized within a six-point range, from ---, denoting the strongest schema negativity to +++, representing the strongest positivity. We hence ended up with annotations such as P+, F--, or B+++ for the scalarized schemas.

The path schema comprised instances of musical elements conceptualized as traversing linear directions, thus causing the conceptualization of motion. As the metaphorical understanding of music moving “through time” (represented in standard Western notation as a “horizontal” progression from left to right) is ubiquitous (Johnson and Larson, 2003), it seemed superfluous to involve this phenomenon as an instance of our path schema: music constantly moves in this sense so paths always operate, and are always positive. Hence we opted for the narrower focus on *vertical* movement only, and specified it as going either “upward” or “downward.” P+ involved instances of unidirectional musical movement comprising the interval of up to a fifth; P++ the same movement of up to one octave; and P+++ such movement exceeding one octave. The force schema in the present paper focused on changes in dynamics only, ranging from F--- for piano pianissimo to F+++ for forte fortissimo. The link schema involved markers of merger or separation between adjacent tones, i.e., changes toward musical patterns with the same articulation type (L--- staccatissimo, L-- staccato, L- portato, L+ unmarked, L++ legato, L+++ legato accompanied by the pedal). The containment schema represented a problem similar to path: if one should interpret it more broadly, as any instance in which a prominent musical element structurally “contains” other elements, one would end up with ubiquitous containment in music, as practically any simultaneous sounding of two or more notes, e.g., in chords, would trigger this schema. We therefore restricted musical containment to non-harmonic tones (passing tones, suspensions and anticipations), as these serve as ornaments, “announcing” the tone after them, thus in some sense “belonging” to this main tone. The containment is negative in music when it is dissonant, i.e., when the embellishing sequence begins and before the main tone ensues, and becomes positive with the consonance, i.e., the sounding of the main tone. The scale of musical containment depends on the number of notes in the non-harmonic cluster: C- stands for a single note, e.g., an appoggiatura, announcing the final note (C+); C*--* is a dyad belonging to the final note, e.g., a very short third- and fifth-degree double-note resolving into the upper tonic (C++); finally, C*---* means three or more notes, e.g., a triplet or an arpeggio, working as constituents of / announcing the final note (C+++). balance schemas have to do with harmonic relations: in B--- two dissonant chords are linked, where one has at least one out-of-key tone, e.g., a dominant and a secondary dominant, with B-- two dissonant chords are linked, remaining in the key, e.g., a subdominant and dominant; while in B- a consonant and a dissonant chord are linked, e.g., the tonic and the dominant. Conversely B*+* involves a linked dissonant and consonant chord, e.g., the dominant and tonic; B++ means that two consonant chords are linked toward the less stable chord, e.g., the major tonic and its parallel minor; finally in B*+++* two consonant chords are linked toward the more stable chord, e.g., the parallel minor and its major tonic.

The musical schemas were annotated by the first author only, since they were extractable from the formal relations in the score and thus objectively verifiable. This was the consequence of the fact that, unlike in language, schemas in music come from structural (thus “grammatical”) relations. In turn, this comprises likely the most important ontological difference between schematicism (and more broadly, semantics) in the two modes of interest in the present paper. Importantly, the annotation followed the schematic rather than music-theoretic structure. In other words, it was carried out schema-by-schema rather than, for instance, measure-by-measure. This helped us not overlook any relevant schemas in portions of the score, as, in effect, we looked for beginnings and ends of our schematic structures which could vary in length considerably. E.g. if an upward passage extended over three bars, that was the level at which the particular schema operated (in such cases, of course, it often included a huge frequency span, so it was annotated as P+++). Conversely, if we had a constant sequencing of staccato and legato or forte and piano articulations, the schema often occupied a single note position. Therefore the criterion for parsing relevant structures was based on our definitions of musical image schemas rather than on any standardized music theoretic chunks, either formal (e.g., measure) or analytical (e.g., phrase).

The linguistic corpus comprised written sources on Beethoven’s piano sonatas by musicians and music theorists: Charles Rosen’s (2002) short companion to the sonatas, Kenneth Drake’s (1994) book on the sonatas highlighting the creative experience while playing them, and Czerny’s (1846/1982) classic didactic piece about the “proper performance” of Beethoven’s works. In selecting the language materials, we opted for pedagogical rather than simply music-analytical texts as we believed their instructive slant would provide both aspects of schematicity that we were interested in: the schemas themselves (as the authors needed to write about the musical material, from ascending scales to disbalancing harmonies) and their scalarization [as the pedagogical purpose required that the authors occasionally provide affectively-laden instructions to the student, e.g., when Rosen (2002, 123) says that Beethoven’s Sonata Op. 2 No. 1 is like Mozart “transported into a new and more violent affective world”]. The three books turn out to be pretty much the only materials of that kind dedicated exclusively to the composer’s piano sonatas (Rosen’s and Drake’s texts as full books, and Czerny’s treatise talking about the sonatas in chapter 2). Yet they also vary in style and focus: Czerny provides short and practical instructions to the young pianist; Rosen is more modern, detailed and addresses the experienced player as well, but his text remains focused on the form of the sonatas and technical challenges while playing them; finally, Drake is much more descriptive, metaphorical and “referential,” often transcending the physical instructions toward a more creative appreciation of the music, as when he says that “The sonata [Op. 2 No. 1], with its self-confident, if gangling stride, moves like a youthful body possessed by a rebellious, indomitable spirit.” (Drake, 1994: 86). Of course, such texts, packed with non-musical associations, are particularly valuable in analyses of image-schematicity and metaphor.

The full corpus of texts on the first ten sonatas from the three books comprised 34,332 words. To prepare this material for annotating the image schemas, the first author of the present paper singled out all phrases in the texts that directly provided a metaphorical description of the music in the sonatas and/or instructions on how to play them. Therefore, the annotation of language units for underlying image schematic structure was performed only on the phrases that contained direct reference to the inherent quality of the music unit and an obviously music-inspired cognitive or affective reaction the particular music sequence has elicited in the engaged percipient, i.e., the author of the text concerned with the sonatas, but not on unrelated phrases. In other words, we looked for direct descriptions of what was “happening” in the music, but at the same time did not restrict our selection to music-theoretic vocabulary alone. For instance, the image schema force would be assigned to “performed with fire and energy” and even to “forces us to remember the main theme,” as in the last case the musical structure functions as a vehicle that incites an emotional or cognitive reaction in the listener. However, forces not directly related to the musical material but rather, for instance, the performance, were not included, e.g., “[the way the passage is played]… gives it psychologically greater force with the public.”

Our such methodological choice excluded portions such as “Were this music of a hundred years earlier, the composer would have relied on other devices” (no reference to the musical material), “Here it would be nonsensical to interpret the *ffp* in the left hand as applying only to the third under which it is written” (no metaphor), or “It must be remarked that, in the following passage, the little note is a long appoggiatura and must therefore be played as a quaver” (direct instruction using only very basic music-theoretic terminology). The phrases that we did select of course also comprised thematically varied materials, but they mostly encompassed statements with three types of referential semantics: (1) those directly describing the musico-theoretic aspects of the sonatas, yet with added attributes (e.g., “broken-chord figures,” “adding the upbeat,” “increasing the forward movement,” “impeding the tempo [...] with an inverted turn”); (2) those relating the performance to affect (e.g., “The character of the whole is decided and vigorous, with brilliant performance,” “[with] great passion, the emotional depth of the Largo overwhelms”), and (3) referential metaphors, strongly relating the music to non-musical, real-world experience (e.g., “one such event soars over broad expanses of many measures and then repeats itself,” “[with] roaring and howling and throbbing [...] the composer burns his musical logic into one’s consciousness”).

Note that the label “metaphor” for the third group alone may be misleading as both music-theoretic notions from group one and affective descriptions from group two can also be labeled metaphorical, e.g. music is inherently neither physically “broken” nor emotionally “vigorous” (cf. Johnson and Larson, 2003; Antović, 2009a). In fact, even many “very basic” music theoretic terms that we excluded from the analysis, such as the “quaver” mentioned above, also have metaphorical origins, and thus may be said to have diachronically had a cross-domain nature as well, which may have gotten lost in the centuries of using the terminology in the West. So it is difficult to draw the lines. Our decision in such borderline cases was to include in the corpus only instances which provide an additional, metaphorical qualification of the theoretical term (such as the “*broken* chord” above).

This selection was then inspected and corroborated by the remaining two authors. In the end, it comprised 2,383 items for analysis. Authors two and three then independently annotated these phrases for the five image schemas of interest. Obviously, unlike in music, where the schemas were traceable by means of formal parameters in the score (e.g., the presence of a slur or a forte symbol), the ascription here was semantic and interpretive, thus potentially subjective. Path was annotated whenever physical or metaphorical paths were referred to in the text (e.g., “extends to the end”) and subsequently specified as going “up” or “down,” when appropriate (e.g., “long skips,” “scale steps”). Likewise, the mention of forces or force-related terms such as pressures, powers, squeezes or burdens was put down as force (e.g., “forces us to remember the main theme,” “energy and will,” “the impulsiveness of youth”). The same applied to link, which encompassed literal or metaphorical instances of attachment or detachment (e.g., “square-shouldered notes,” “separates the youth”). balance included expressions referring to the preservation or loss of stability [e.g., “maintains the seriousness,” “a revolutionary (move)”] while containment targeted conceptualizations in which literal or metaphorical 3D objects with volumes and borderlines with the outer world were invoked (e.g., “the most compressed,” “the first beat is always blank”). The schemas were again scalarized with three positive and negative levels, as in the case of music above. As a result, the annotations comprised either individual schemas (e.g., “as though clasping hands with the composer in life”: <L+++>, “powerful”: <F+++>) or, much more commonly, their combinations, i.e., schematic complexes [e.g., “(this) shows changes of momentous importance”: <P++ > <Spec> < up> < down> < B- > <F++>]; “throw the listener off balance”: <C+ > <B-->; “following the fortissimo in the preceding measure”: <F+++ > <P+ > <Spec> < forward>; “the spinning out of simple ideas” (<C+ > <P+ > <Spec> < forward> < Spec> < out>).

Upon the end of the procedure, the two separate ratings were compared. Cohen’s Kappa was 0.72, indicating solid agreement, in particular having in mind the substantial number of comparisons. Cases of disagreement were subsequently resolved by the majority vote among the three authors, providing the final annotated list.

### 2.1 Data analaysis

As the size of the two corpora (musical and linguistic) was different, and also because the size of the sub-corpora within the modes (e.g., individual movements of the same sonata) varied, we could not use absolute sums of schemas for comparison. Rather, opting for the standard procedure in linguistic corpus research, we employed “schema densities”: we calculated the average number of schemas per 1,000 words (language) and per 1,000 notes / measures (music), to come up with averages for comparison. Not to confuse non-linguist readers we shall use the term “frequency” instead of “density” in further text, yet we add the caveat that by this we mean the averaged frequency, that is “frequency per 1,000 units.” Naturally, the choice of notes and measures in music was somewhat arbitrary as there is no direct equivalent of “words” in musical structure (though in language science, too, researchers often avoid “the word” as a concept due to serious problems defining it, e.g., Palmer, 1976: 37–42). In that sense, coming up with, e.g., 10 schemas per 10 words was not directly comparable to, e.g., 10 schemas per 10 notes or measures. Yet, as will be seen in further text, this incommensurability was not a problem for our general analytical strategy: counting the frequencies of items within the modes and then comparing the resulting prevalences across the modes. Namely, our goal was not to directly compare the musical and the linguistic frequencies or averages (across the modes), as there certainly was no one-to-one correspondence between the musical structure and the texts about the music (otherwise, the text would have had to describe the music measure by measure, if not note by note, which was of course not realistic). What we did hope to find, though, was convergence in more general tendencies by comparing across the modes the *results* of the comparisons made within the modes (e.g., if there were more positively-valenced schemas in the music, was this the case with the language as well?). In other words:

1. Our analysis first determined the overall number and frequency of schemas in the musical and linguistic corpora. This was done in order to learn whether, as expected, due to their structural rather than interpretively-semantic nature, the musical schemas would outnumber the linguistic ones.

After this, we employed four main calculations.

1. We compared the distributions of the schemas per scalarity level. More precisely, we wished to ascertain whether schemas with just one scalarity symbol (just one minus or plus sign, the “normal,” “weak,” “unmarked” strength) outnumbered those with scalarities “two” and “three” (stronger, pronounced or very pronounced scalarity, again either negative or positive) in either language or music. We achieved this by means of one-way ANOVA tests with Tukey’s HSD post-hoc comparisons. We hoped any observed tendencies would be mirrored in both modes.
2. We compared the ratio of positive and negative valences (taken as a whole) for each of the five schemas, in music and then in language. We did this so as to see whether the overall ratio of schema positivity and negativity would converge in music and language. This was achieved by means of Student’s t-tests conducted per schema per mode (e.g., positive vs. negative F schemas in music, and then in language, separately). Again, we hoped any observed tendencies would apply to both language and music.
3. We compared the ordering of the schemas by frequency per sonata in music and in language, by positive and negative valences, respectively. The goal of this procedure was to ascertain whether the internal hierarchies of schema frequency were the same or different in the music of the sonatas and the texts about them. To achieve this, we first calculated the grand averages of schema frequencies (means and standard deviations, e.g., the mean value of F+ through ten sonatas) and then calculated 95% confidence intervals for each value obtained in such a way. We then looked at any overlaps among the confidence intervals to determine which schemas seemed to be more, and which less frequent in music and then in language. Finally, we compared this final result in the two modes.
4. In the most important analysis, we calculated the average frequencies for all six scalarity levels per each schema in music and language, first per sonata and then, in a most detailed manner, per movement. Then we looked for any correlations between the distributions of these averages, in music and language, by means of Pearson’s correlation coefficients. We hoped for significant and high correlations, suggesting that the overall distribution of the three negative and three positive intensities per schema in both music and language would converge. In turn, this would support the main thesis of the study - that the inferred frequencies of five targeted schematic structures in the music served as primes for the three authors to end up using comparable frequencies of linguistic schemas in their texts.

## 3 Results

1. The absolute number of schemas in the linguistic corpus was 13,025 (per 34,332 words total). In relative terms, this amounts to the frequency of 397 schemas per 1,000 words. The musical corpus had 19,164 schemas total (per 89,261 notes or 6,621 measures). In relative numbers, this equals the frequency of 214 schemas per 1,000 notes or 2,895 schemas per 1,000 measures. Naturally, since practically any chord played in the sonata includes more than two pitches, “one note” in the scores was hardly the equivalent of “one word” in the books, in any context imaginable. As stated above, no comparison between “the word” and any musical structure really makes sense (perhaps one letter or one syllable are a slightly better match for one note, though again there is no substantial, structural parallel to motivate even such a connection). For this reason, we focused on the sheer temporal length of items as a possible constituent for comparison. Viewed that way, at least in terms of the number of minimum units inherent to the pattern (two to about twenty phonemes or pitches), “the measure” in music is probably a much more adequate comparison for “the word” in language. Viewed that way, i.e., per temporal units of roughly equal length, musical schemas were approximately 7.29 times more frequent than linguistic ones.
2. In terms of schema intensities, in music, one-way ANOVA tests showed a significant effect for positive schema valences with intensities one, two, and three, respectively: *F*(2, 147) = 41.66, *p* < 0.0001. Tukey’s HDD test revealed that the mean score, i.e., the frequency of schemas per 1,000 notes, for the + condition (*M* = 16.96, *SD =* 11.59) was higher than frequency for the ++ condition (*M =* 9.36, *SD =* 10.73), which was still higher than the frequency for the +++ condition (*M = 0*.32, *SD = 0*.53). The same tendencies were shown with regard to negative valences of musical schemas: *F*(2, 117) = 31.98, *p* < 0.0001. Tukey’s HDD test again showed the highest frequency for the - condition (*M* = 14.36, *SD =* 12.27), which was higher than the frequency for the -- condition (*M =* 6.44, *SD* = 6.42), which was higher than the frequency for the --- condition (*M* = 0.09, *SD* = 0.19). All pairwise differences were significant (*p* < 0.01).

In language, there was likewise a significant effect for the frequencies per 1,000 words of positive schema valences with intensities one, two and three [*F*(2, 147) = 65.11, *p* < 0.0001]. Upon using Tukey’s HDD test we found that the frequency for the + condition (*M =* 23.09, *SD =* 15.97) was higher than the frequency for the ++ condition (*M =* 5.91, *SD =* 5.01) and the frequency for the +++ condition (*M =* 2.20, *SD =* 2.48) (*p* < 0.01). The difference between frequencies two and three was, however, not significant. The same tendency was registered with negative schema valences in language. One-way ANOVA revealed a significant effect [*F*(2, 117) = 44.43, *p* < 0.0001]. The frequency for the - condition (*M =* 4.25, *SD =* 2.57) was higher than the frequency for the -- condition (*M =* 1.52, *SD =* 1.31) and also than the frequency for the --- condition (*M = 0*.68, *SD =* 1.06) (*p* < 0.01). The difference between frequencies two and three was not significant.

1. In music, the schema frequency for positive F schemas (*M =* 16.74*, SS =* 112.50) was significantly higher than the frequency for negative F schemas (*M =* 9.37, *SS =* 45.01): *t*(18) *=* 5.57, *p* < 0.0001. Likewise, the frequency for positive L schemas (*M =* 44.43, *SS =* 2018.27) was significantly higher than the frequency for negative L schemas (*M =* 25.75, *SS =* 500.22): *t*(18) = 3.53, *p* < 0.005. Interestingly, schema frequency for positive B schemas (*M =* 25.86, *SS =* 594.54) was significantly *lower* than the frequency for negative B schemas (*M =* 40.02, *SS =* 2548.75): *t*(18) = − 2.4, *p* < 0.05. There were no differences in the frequencies of positive and negative C schemas, nor in the frequencies of P schemas specified as going “up” and “down.”

In language, the schema frequency for positive F schemas (*M =* 34.28*, SS =* 426.58) was significantly higher than the frequency for negative F schemas (*M =* 6.39, *SS =* 29.24): *t*(18) *=* 12.39, *p* < 0.0001. Likewise, the frequency for positive L schemas (*M =* 23.24, *SS =* 263.44) was significantly higher than the frequency for negative L schemas (*M =* 9.56, *SS =* 100.08): *t*(18) = 6.81, *p* < 0.0001. Here, positive C schemas (*M =* 26.26, *SS =* 313.05) prevailed over negative ones (*M =* 3.38, *SS =* 11.83): *t*(18) = 12.04, *p* < 0.0001. There was no significant difference in the frequencies of positive and negative B schemas, nor in the frequencies of P schemas specified as going “up” and “down.”

1. The grand means, standard deviations, and 95% confidence intervals, for the frequencies of positive and then negative schemas, in music and then in language, are given in Table 1.

**Table 1:** Grand means, standard deviations, and 95% confidence intervals for frequencies of schemas per unit in music and language.

Schema	Music			Language		
	*M*	SD	95% CI	*M*	SD	95% CI
F-	9.37	2.24	7.98–10.76	6.39	1.80	5.27–7.51
P-	17.41	6.98	13.08–21.73	6.26	2.78	4.54–7.98
L-	25.75	7.45	21.13–30.37	9.56	3.33	7.50–11.62
C-	8.46	3.80	6.10–10.81	3.38	1.15	2.67–4.09
B-	40.02	16.83	29.59–50.45	6.46	4.11	3.91–9.01
F+	16.73	3.53	14.54–18.912	34.28	6.88	30.02–38.54
P+	17.81	7.27	14.54–18.92	9.00	2.25	7.60–10.39
L+	44.43	14.97	35.15–53.71	23.24	5.41	19.89–26.593
C+	25.86	8.13	20.82–30.90	26.26	5.90	22.60–29.92
B+	22.74	14.76	13.59–31.89	5.07	2.15	3.74–6.40

The criterion for the ordering of schemas by strength in any category (music, language, negative, positive) was the lack of overlaps in 95% confidence intervals. This allowed us to claim that the ranges of possible deviation from the mean value did not affect the separation of the categories (schemas by frequency), with the significance level of *p* < 0.05 (Table 2).

**Table 2:** Ordering of schemas by frequency, with 95% confidence intervals.

Valence	Music	Language
Negative	B- > P-, F-, C-; L- > F-, C-; P- > F-, C-	F-, P-, L- > B-, C-
Positive	L+ > P+, C+, B+ C+ > F+	F+ > P+, L+, C+, B+; C+, L+ > P+, B+; P+ > B+

In other words, in music, negative balance is stronger than negative path, force, and containment. Negative link and path are both stronger than negative force and containment, but are not different from one another. In language, negative force, path and link are stronger than negative balance and containment, but no finer-grained claims are possible.

Likewise, in music, positive link is stronger than positive path, containment and balance, while positive containment is stronger than positive force. In language, positive force is the strongest of all schemas. Positive containment and link are stronger than positive path and balance, and finally positive path is stronger than positive balance (making positive balance in language the weakest of all schemas).

1. In the correlational analysis, our intention was to preserve the ontological unity of our five schematic categories (where “a force is always a force”), yet still account for the differences in valence and arousal (where piano differs from forte). Thus, we wanted to be able to compare how the frequency, valence, and strength of a particular schema (for instance, force) varied by the sonata and movement, in music and language alike. To achieve this, we opted for *weighting* the schema frequencies. In other words, depending on the valence and scalarity, we multiplied each frequency by a coefficient: --- by 0.3; -- by 0.6; - by 0.9; + by 1.2; ++ by 1.5, and +++ by 1.7. The intention was, of course, to increase the final values for positive scalarity (thus multiplication by more than 1) and decrease them for negative scalarity (thus multiplication by less than 1). The step of 0.3 between the adjacent ranges was calculated on the basis of our prior classification of musical P schemas (Antović et al., 2023) and adopted in the present paper. To reiterate, P+ implies a jump of up to one fifth, P++ of up to one octave, and P+++ of more than one octave, typically up to a twelfth. Therefore, the average range between adjacent values is a third, which amounts to the frequency increase of 5/4 or 0.25. We rounded this off to 0.3 to avoid no weighting (multiplication by 1) with the scalarity of +. We then calculated Pearson’s correlations for all six intensities per schema, in two steps: first, the grand averages per sonata (6 scalarities x 10 sonatas = the sample of 60 items per schema) and second, the averages per movement (6 scalarities x 35 movements = the sample of 210 items per schema). One exception was that of P schemas, for which we had the binary choice of “up” and “down” specification (so the sample amounted to 30 items per sonata, and 105 per movement).

By sonata, results of the Pearson correlation indicated a significant large positive relationship between the weighted frequencies of musical and linguistic F schemas: *r*(58) = 0.562, *p* < 0.0001; P schemas: *r*(28) = 0.865, *p* < 0.0001; C schemas: *r*(58) = 0.639, *p* < 0.0001; and B schemas: *r*(58) = 0.772, *p* < 0.0001; and a significant medium positive relationship between the weighted frequencies of musical and linguistic L schemas: *r*(58) = 0.352, *p* < 0.01.

By movement, the granularity of analysis was the highest, as it allowed us to also compare separate musical sections by type (e.g., faster and more vigorous separately from slower and more peaceful movements). It also increased the sample size, but of course resulted in the reduction of the effect, which was still strong enough for reaching conclusions. Here, Pearson correlations revealed a significant large positive relationship between the weighted frequencies of musical and linguistic P schemas: *r*(103) = 0.73, *p* < 0.0001; and B schemas: *r*(208) = 0.508, *p* < 0.001. The relationship was medium, but still statistically significant, with the remaining three weighted average schema frequencies: F schemas: *r*(208) = 0.420, *p* < 0.0001; L schemas: *r*(208) = 0.348, *p* < 0.0001; and C schemas: *r*(208) = 0.441, *p* < 0.0001.

Overall, there were few or no outliers impeding the use of the Pearson test and the results may be interpreted as valid.

## 4 Discussion

The goal of the present research was to test whether image schemas operate in a similar form across cognitive modes. In particular, we wished to ascertain whether the abundant (or not) employment of particular schemas in music primes the cognizer to use an equally abundant (or not) share of the same schemas in language about this music. To investigate this, we studied whether five types of such schematic constructs (path, force, link, containment, and balance) appear in commensurable forms in both classical music and language about this music.

“Commensurable” remains the key term to define, as looking for full equalities in the two modes (for instance, in terms of identical numbers or combinations of schemas per unit) does not appear realistic. Namely, music and language have been compared quite successfully on the “formal,” “grammatical” and “syntactic” levels in recent decades (Lerdahl and Jackendoff, 1983; Rohrmeier, 2011; Zbikowski, 2017, *inter alia*). On the other hand, even though they have a very long historical tradition, from Plutarch (Pöhlmann, 2011), over Rousseau (1781/1998) and Darwin (1874/1997), to Susan Langer (1957) and Cooke (1959), to, today, Cross (2009), semantic parallels typically fail. Music hardly has denotations (except in trivial cases of imitating real-world sounds), it does not provide “senses” or “references” in any comparable way to language, and even when it does seem to generate the same constructs as in language - e.g. connotations - it turns out that, unlike their linguistic counterpart, these are, at best, imprecise. Therefore, theorists usually insist that musical meaning is of a very different *kind* from that found in language (Jackendoff, 2009). In fact, even recent theories that support the case for a musical semantics approached from a meta-linguistic perspective (Schlenker, 2019; Antović, 2022) remain rather cautious about all-out parallels between units of meaning in music and language. Rather than insisting on such surface-level structural constructs (e.g., in the assumption that, analogously to a linguistic lexeme, one may postulate a musical “museme,” Seeger, 1960), it looks much more useful to go deeper into the abstraction, and propose connections that enable the cognitive system to come up with those constructs in the first place. This process, then, requires “zooming out” from surface phenomena in order for analogies to work better. What might be shared by music and language semantically, from such a perspective, is underlying cognitive *processes*, such as the parsing of stimuli on the basis of gestalt perception, ascribing affect to the succession of less and more tense formal phenomena, metaphorization, the ability to create conceptual blends, to name but a few.

Image schemas (which often serve as the basis of metaphors) turn out to be a good test case for an approach to multimodal semantics which targets such “underlying” cognitive phenomena. There are at least two reasons for this. First, schemas are not units of meaning in themselves, but rather only *help* the cognitive system better *ground* meanings. A musical succession from C4 to C5 or the expression that “one is climbing the ladder of success” does not realize paths in any inherent way. Cross-modal correspondence researchers still debate the origins of these connections. For instance, “statistical” approaches think that high tones simply tend to come from elevated sources (Parise et al., 2014), “semantic” ones believe that the use of particular terms in a language favors some options - such as verticality, over others - e.g. thickness (Martino and Marks, 1999), while “emotional” proposals look for a matched “hedonic valence” of the stimuli from the two modes (Spence, 2020). Closest to our view seems to be the “amodal” position (Walker, 2016), which also branches into several sub-variants: e.g. that the properties of the stimulus are picked up by different senses (linguists would call this “multimodal,” e.g., Bateman et al., 2017); or that amodality serves to complete missing perceptual information, e.g., when a part of the stimulus is occluded. Our use of the term belongs to yet a third group, in which perceptual stimuli trigger formal representations lacking any modality, which play a role in the higher processing of sensory information (for an excellent recent review of positions on amodality, see Spence and Di Stefano, 2024). From this last perspective, the high-low conceptualization of musical pitches in fact corresponds well to just about any perceptual opposite (e.g., bright-dark or female–male). When creating the correspondence, one does not focus on the individual qualities of the two tones, but rather on their interrelation, which is structural and abstract.

One reason for which we embrace the amodal view is the fact that we have our own empirical evidence to support it (Antović et al., 2020). Another is that current work in neurosemantics indicates that, after a long period of belief that meanings are grounded in “modality specific cortices,” recent data from multisensory research suggest the time has come for a “property specific and modality-invariant turn,” even in the construction of concrete word meanings, which are most liable to “grounded” approaches (Calzavarini, 2024: 815). Based on such an epistemology, there might be nothing particularly vertical about the sequencing of pitches or even less about the improvement of one’s social status. It is the cognitive system that later ascribes the, probably embodied and experiential, sense of “elevation” to both of these constructs, thus “specifying” one particular option for conceptualization amongst a number of amodally-constrained possibilities. First, this way their meaningfulness seems more natural, as the connection between “going up” and “feeling good” is probably made very early in childhood (Mandler, 1992). Second, such a schematic grounding makes these meanings easily extendable in further contexts. Thus one can expect that both types of ascent should at one point reach the top (of the musical structure or of the social success), which would open up the venue for further creative conceptualizations and interpretations. Due to this first function they perform, schemas allow deep, structural parallels between musical and linguistic semantics, as proposed by our group before (Antović, 2022; Antović et al., 2023) and pursued further in the present paper.

The second important way in which image schemas operate as a “deeper-level” cognitive mechanism motivating constructs in either language or music has a directly opposite function: to suggest how musical and linguistic meanings *differ*. Though the distinction is not absolute, musical meaning is largely *iconic*, while linguistic meaning is primarily *interpretive* (cf. Bierwisch, 2009). In other words, most “semantic” interpretations of music are derived directly from musical *form*, and then developed by means of creating analogies, metaphors and conceptual blends from this form, probably in concentric circles of recursion (Antović, 2022). So, for a Beethoven excerpt to be interpreted as “nervous” or “intense,” one would first need to hear a lot of fortes, probably in a quick tempo, staccato articulation, and, perhaps, the minor mode. Up and above such “intensity” one can of course construct meanings that look more interpretive, e.g., in claiming that Sonata Op. 2 No. 1 “asserts the young composer’s individuality” […through…] “unprecedented excitement” (Rosen, 2002: 123). Yet the basis for all these verbalizations remains - the physically perceived loudness. In language, we find the completely opposite situation: there is arguably no perceived intensity in the way the terms “intense,” let alone “assert”(ed) or “excitement” *sound*. So meaning does not derive from form (at least not directly) and needs to be constructed on the basis of interpretation. Just like with interpretation in music, which comes in later and remains optional, we find the same process, yet moving in the opposite direction, in language: once the basic interpretive process has finished, some sound-to-semiosis correspondence may come in later, e.g., in alliterative poetry. So the humming undertone of a “busy bee buzzing” may indeed strengthen our understanding of the way this insect flies. Yet the *primary* meaning of the expression derives from our knowledge of the words’ denotations and the logical form beneath the syntactic structure.

Therefore, it is this interplay of “parallels and non-parallels” (Jackendoff, 2009) that vouches for the employment of schemas as a test case for interesting phenomena underlying music and language perception. In both language and in particular music, image schemas are typically studied in qualitative analyses (Antović, 2018). In contrast to this, the present study attempted to approach the problem of “schematic” convergences, or lack thereof, from a quantitative standpoint: by analyzing two sizable corpora and counting instances of schemas within them.

It has turned out, indeed, that the difference in structure is best seen with regard to the sheer number of schemas per unit, which relates to our hypothesis 1. This is shown in observed schema frequencies. As mentioned in the results section, and in particular due to the structural discrepancy between linguistic and musical *constructs* explained above, it was quite hard to decide on the musical equivalent of the linguistic “word.” Musical tones, chords, phrases, or “sentences” have neither the morphosyntactic compositionality nor the denotational meaning equivalent to words, so again no structural comparison was justifiable. In the end, we opted for the “measure” due to a more general cognitive constraint it imposes on the listener: namely, size. In English, words range anywhere between one and about twenty speech sounds (the longest word apparently has forty five phonemes), while normal speech pace allows for 110–150 words per minute. It turns out that the musical measure typically contains between one and about twenty notes, too, while Beethoven’s Sonata No. 2 Op.1, for instance, requires the meter of about 108 beats per minute.

This comparison has revealed that there are over *seven times* more schemas in music than in language per unit. This, of course, matches our expectations. If a construct is derived from form alone, and requires no finer-grained semantic processing (i.e., interpretation), it will likely occur much more often in a cognitively-demanding, temporally-evolving structure. So iconic schemas are much more frequent than interpretive ones, fortifying the substantial difference between musical and linguistic meaning.

More complex cases of “both parallels and non-parallels” were seen in our hypotheses 2, 3, and 4. The second one related to overall schema strengths, i.e., arousal they invoked in the listeners / writers of the three texts. Here we compared the ratio of level one, two and three intensities (both negative and positive) of schemas in the musical and linguistic corpus. Following standard linguistic terminology, we have taken “level one” schema strengths (instances of schemas with a single plus or minus sign) to be “unmarked,” i.e., typical cases (e.g., a jump of a third in music, turning from pianissimo to piano; or the expression in language that the music “transfers [itself]” between registers: <F+ > <P+>). “Level two” and “level three” schema strengths were, on the other hand, “marked,” i.e., particularly pronounced (e.g., a jump of a sixth in music, turning from piano to fortissimo, or the expression in language that the music “constantly augmented power”: <F++ > <P++>).

The result here was that, in both modes, unmarked schema strengths stood out. It turns out that, even in Beethoven’s music, which is apparently much more vigorous than that of his predecessors, “normal” intensities of musical elements (i.e., relatively small intervals, changes in dynamics or articulation) prevail. In turn, this is mirrored in the verbal expressions about this music in our three books of interest. Such a result was somewhat surprising, as we expected to find more marked intensities, especially those of “level two,” in both corpora. Yet this expectation was likely a consequence of the cognitive system’s tendency to misinterpret quality as quantity. In this sense, just as one erroneously concludes that everyone in a city must be rich because one has noticed several expensive cars on the street, one equally concludes that all of Beethoven’s music is very loud because of an occasional burst of prominent forte sections. So we may have inadvertently disproved a common misconception with this result. Our current assumption is that, for more prevalence of marked schemas, one would need to analyze more recent musical pieces, starting perhaps with the High Romantics, such as Chopin or Rachmaninoff.

The predominance of “level one” intensities in the two corpora now serves as the first proof of parallels between linguistic and musical schematic meanings: those relating to relatively low overall arousal. On this coarse-grained level, the two modes seem to be quite similar to one another. A difference, however, emerges in the immediate next result. Namely, while in music, “level two” and “three” intensities also significantly differed, in language they did not. This again likely comes from the nature of the two types of semantics. The fact that musical meaning derives from form allows for easier finer discretization of schema scalarities. In other words, the fine distinction between pitch leaps (of up to a fifth, an octave, or more than an octave) is immediately seen in the score, or heard in the performance. On the other hand, in linguistic expressions, fine distinctions (e.g., between “moving” and “extending the motion”) are more interpretive, and thus much more difficult to classify.

With regard to our hypothesis three, the results also vouch for both parallels and non-parallels, yet seem to gravitate more strongly toward the “parallel” end. The question here related mainly to valence, i.e., which polarity was more common with particular schemas, in either mode. e We looked at the ratio of positive and negative valences per schema to see if the prevalence of one or the other in music would be found in language, too. If so, this would mean that, for instance, more linked than unlinked articulations (e.g., slurs as opposed to staccato dots) in music would be accompanied by more linked than “disconnected” semantics (e.g., “reaching” as opposed to “detachment”) in the three texts, too.

Three out of five schemas showed equal polarities of the kind defined here. With regard to force and link, there were significantly more positive than negative schema valences in both language and music. In terms of path, there were no differences between sequences going “up” and “down” in either music or language. Containment showed significantly more positive valence in language, while in music there were no differences. Finally, negative balance prevailed in music, while in language there were no differences.

It turns out, therefore, that “stronger” (louder…) music was accompanied by linguistic expressions referring to strength; that more “linked” articulation in music resulted in more imagery of things being connected or interrelated in the texts; and finally that both musical sequences and linguistic phrases about them contained a relatively even ratio of (descriptions of) movement upward and downward. Once again, this suggests that the text authors unconsciously “copied” the rich structure of the musical schemas in their linguistic output, likely because the inferred musical schemas primed them to do so. The difference in containment is likely not a real difference, either. Namely, as explained in the analysis section, we may have defined musical containment rather conservatively prior to analyzing our corpora. First, we included in this category only (relatively rare) non-harmonic tones, such as anticipations, and then we additionally divided most containment sequences as having a negative (dissonant, with the non-harmonic tones) and positive (consonant, with the resolving tone) component. This may have, first, dramatically reduced the actual numbers of containment schemas in music since (positively valenced) containment likely represents *any* situation in which, in addition to the most prominent musical tone (e.g., the lead in the soprano), a metrical slot also contains other tones (e.g., the harmonic accompaniment). In that sense, every chord in the sonatas is an instance of containment. Consequently, the number of positive schemas of this type rises exponentially, resulting in the same situation as in language (many more positive than negative containment valences). Additionally, marking dissonant passing or ascending tones as C- and consonant resolving tones as C+ was essentially based on the criterion of harmonic stability. In turn, this may have confounded containment and balance schemas, which represents a methodological concern. In all, the result for containment is likely a consequence of our very conservative treatment of this schema in music, which should be addressed in future research. The only real difference between the two modes in this respect, therefore, remains related to the balance schemas. This is likely a real non-parallel in valence, since music is inherently dependent on the experience of (especially harmonic) disbalance, where dissonant chords and sequences prevail throughout the musical flow, and resolutions, if any, ensue only occasionally. This stands in sharp contrast to language, where semantic stabilities and instabilities seem to be much more evenly deployed.

Results related to hypothesis 4 also provide an interesting combination of parallels and non-parallels. The key question here was sequencing by frequency: what did the final “hierarchy” of the five schemas look like and was it the same in both modes? Here we ordered the five schemas by frequency in the corpus (again viewed as frequencies per 1,000 words or notes, i.e., means and standard deviations). The goal was to ascertain whether the hierarchies for music and language would be the same or different. As expected, differences between each adjacent pair were not statistically significant, so we resorted to 95% confidence intervals to hypothesize with some certainty which *groups* of schema frequencies were likely more dense than others.

The result here is liable to interpretation and may be defended from both ends. On the “non-parallel” side, at the first glance, the ordering is clearly different. This is almost certainly a consequence of the fact that we mentioned several times so far – that the linguistic material never describes music in a one-to-one manner, but often gives “summary” statements about schematic complexes that might repeat in the music dozens of times. It turns out, therefore, that negative balance in music is the strongest, followed by links and paths ranking second, and then forces and containments ranking third. In language one can only claim that negative forces, paths and links are stronger than balances and containments. In terms of positive valences, musical links are the strongest, while paths, balances and containments are roughly equally common, with only containments being more frequent than forces. In language, positive forces are the strongest and balances the weakest, with the remaining schemas roughly falling in the same range, but still with containments and links being stronger than paths.

More toward the “parallel” interpretation, if one looks at such hierarchies from a yet broader perspective of “strong” and “weak” (more and less frequent) schemas, one returns to the impression that the musical structure primed the writers to use the “strong” schemas more often in the texts. So one may conclude that the prominent negatively-valenced schemas in music are balance, link and path, and in language force, link and path. Prominent positively-balanced musical schemas are link and containment, and linguistic ones - force, link and containment. Viewed that way, language and music seem to share the overall prevalence of negative links and paths and positive links and containments. What separates them is the widespread presence of negative balance in music, which we discussed in the previous point, and, somewhat surprisingly, the much more conspicuous positive force in language. It turns out, therefore, that there were more positive forces in the language material (descriptions involving power, energy, weight, or strong, squeezing, pressurizing, etc. activities) than there were pitch successions involving transition to more forte dynamics in the music itself. This is an interesting result that requires further consideration.

Finally, the result of hypothesis 5 has emerged as a consequence of the most complex and finest-grained analysis. Importantly, it provides the strongest support for the main hypothesis of the paper: that the inference of schematic structures in the music functions as a prompt, if not a full prime, for the text authors to use them in their linguistic descriptions as well. Here we weighted the six scalarities per schema (2 valences, positive or negative x 3 scale levels per valence) and then considered each of the five schemas with such recalculated values as a single category. Then we looked for any correlation between each of the five schema pairs for music and for language. By sonata, this gave us 60 items per category (except for path, where the number was 30, see the Results), and resulted in all five significant correlations, four strong (force, path, containment and balance) and one medium (link). By movement, we ended up with 210 items per category (105 for path, see the Results), and discovered that all five correlations were significant, of which two were strong (path and balance) and three medium (force, link and containment).

This is the strongest result that defends our idea of a “commensurable” schematic structure in music and language. Namely, it shows that, even though the absolute numbers of schemas differ substantially, their relative weighting in the two modes, through six levels per schema, viewed by the sonata, and then by the movement, mirrors one another. In other words, when the frequency of any schema category (force, path, link, containment, and balance), in either valence or any arousal level (e.g., P++), rises or falls in the music, it equally tends to rise or fall in the language, too. In this sense, image schemas in texts about the Beethoven sonatas appear to reflect image schemas in Beethoven’s music, even though the description in no way follows the musical structure one-to-one.

Of course, the most well-known issue surrounding correlations is that they cannot be interpreted causally. In other words, it might be the case that rises and falls in schema frequencies in one mode have nothing to do with the corresponding rises and falls in the other. This is a good warning when one compares the achievement of different participants in experimental designs but does not really affect our conclusions here. Namely, the texts that we compared with the sonatas were *purposefully about those sonatas.* It therefore stands to reason to at least hypothesize that the correlations should have something to do with the texts’ topic, i.e., the music that we equally subjected to the schematic analysis. In fact, to control for this aspect, we went even further, by selecting for analysis portions of those texts that most directly and indisputably described the musical structures in the sonatas. Yet this process could not be exclusive, again due to the fact that the texts did not describe musical structure in a one-to-one manner (e.g., phrase by phrase) or focused on music-theoretic aspects alone. Therefore, in addition to portions obviously describing the musical form (e.g., “a rising sequence”) the analyzed text often contained expressions with the targeted image schemas which did not talk about the scores in the sense of employing technical, music-theoretic terms (e.g., “the crescendo restores unpretentious lyricism” or “the trio makes a rough contrast”). These expressions, too, were highly image-schematic and included in our pool, suggesting an apparent discrepancy between the two modes, which again derived from the different nature of musical and linguistic semantics (iconic vs. interpretive). In fact, it was totally conceivable that a movement containing dozens of added fortes be described by a single sentence with a force schema, such as “This section is much louder,” where the rest of the text would be all about paths and containments. The most interesting result, in our view, is that nothing remotely similar happened. Rather, whether or not they described the music-theoretic aspects of the score directly, schema frequencies rose and fell in the texts as they did in the music. This supports our hypothesis that there was a priming effect (on the book authors) sparked by their (unconscious) inference of the schematic structure from the music. And this, of course, extends beyond Beethoven. Time and again, one finds texts targeting so well the image-schematic structure of musical pieces: e.g. iconic constructs inferable from madrigals by Monteverdi (Georis, 2005) or Lucca Marenzio (Strykowski, 2017), often related to height (a tendency that would be picked up by most Baroque composers). There are further historical instances of text painting supporting our case here, as in a staccato suggestive of knocking in Bach’s Ich klopfe an, from the Advent Cantata (Zbikowski, 2008), to Rachmaninoff’s choral works, such as the Liturgy of St. John Chrisostom (Riazanova, 1999). Likewise, one can find excellent examples moving in the opposite direction, where a literary text schematically inspired a musical piece. For instance, in Vaughan Williams’ the Lark Ascending the constant circular and upward paths in the score are directly inspired by the linguistic description of the bird soaring upward and circling in the sky in George Meredith’s poem of the same name (Antović, 2021). The task for future work, therefore, is to make our concept analysis more generally applicable by involving a broader pool of musical styles and examples. Simultaneously, further alignment with cognitive linguistic methodologies and interests (e.g., force dynamics or construction grammar) would be beneficial.

The ultimate question is whether the phenomena considered in this study are perceptual or metaphorical (thus conceptual). While any opinion remains speculative, our conclusion is that they lean on the conceptual end, even though the dichotomy may turn out to be a continuum. In fact, the idea that historical dualisms in music theory (e.g., form/content, intramusical/extramusical, perceptual/conceptual) should be interpreted as just extreme points on a scale is one of the main tenets of the first author’s multilevel-grounded musical semantics (Antović, 2022). It appears to us that the meaning generation process proceeds in piecemeal fashion. The perceptual properties of the music (e.g., many added fortes and extreme pitch changes) are not conceptual at the outset, where they are perceived as little more than ineffable changes of energy. Yet already at the next level of appreciation, the cognizer cannot help but relate them to elementary spatial and haptic sensations, which motivates schematicity and becomes, in the terminology of cognitive linguistics, “pre-” (Johnson, 1987) or “proto-conceptual” (Sinha, 1999). This halfway conceptualization is then used as a (subconscious) prompt by the cognitive system to include such registered image schematic complexes in the linguistic description as well. It is here that the schematic descriptions (a) proliferate - hence the correlations in our analysis, but also (b) become fully and undoubtedly metaphorical, whether they talk about the musical form (“the melody goes up”) or about the broader musical experience (“the forceful impact of the section”). The fact that they are expressed linguistically, of course, only adds to their metaphoricity. So it appears that the duality of perception and (metaphorical) conception is ultimately a scale, and should be viewed as such especially in iconic semantic generation, such as the one arising from music listening.

In conclusion, our results suggest that a cognizer infers an image-schematic structure in the music he or she listens to, and that this series of inferences may serve as a prime for the same cognizer to use these schemas with a corresponding frequency when talking about this music. There are, of course, differences between the schematic structures in the two modes, as well: music boasts many more image schemas than language and detailed hierarchies of schemas by frequency in the two certainly differ. Yet, at the same time, both modes prefer unmarked schema scalarities; the relationship of positive and negative valence for the schemas mostly converges; some strongest and weakest schemas are shared by the two modes; most importantly, significant correlations, which importantly take into account the valence and scalarity of each schema, are absolute. We hope that this result will encourage further discussions in the related fields of musical cross-modal correspondences, music and language comparisons in cognitive science, and image schemas and metaphor in cognitive linguistics.

## Data Availability

The original contributions presented in the study are included in the article/supplementary material, further inquiries can be directed to the corresponding author/s.
